# Transvenous Pulmonary Chemoembolization and Optional Microwave Ablation for Colorectal Lung Metastases

**DOI:** 10.3390/jcm12103394

**Published:** 2023-05-10

**Authors:** Thomas J. Vogl, Lars Hammann, Hamzah Adwan

**Affiliations:** Department of Diagnostic and Interventional Radiology, University Hospital, Goethe University Frankfurt, Theodor-Stern-Kai 7, 60590 Frankfurt, Germany

**Keywords:** colorectal lung metastases, TPCE, MWA

## Abstract

(1) Purpose: To evaluate tumor response and survival of patients with colorectal pulmonary metastases treated by transvenous pulmonary chemoembolization (TPCE) alone with palliative intent or TPCE followed by microwave ablation (MWA) with potentially curative intent. (2) Material and methods: A total of 164 patients (64 women and 100 men; mean age: 61.8 ± 12.7 years) with unresectable colorectal lung metastases and not responding to systemic chemotherapy, who either received repetitive TPCE (Group A) or TPCE followed by MWA (Group B), were retrospectively enrolled. The revised response evaluation criteria in solid tumors were used to assess treatment response in Group A. The oncological response in Group B was divided into local tumor progression (LTP) and intrapulmonary distant recurrence (IDR) after MWA. (3) Results: The 1-, 2-, 3-, and 4-year survival rates were 70.4%, 41.4%, 22.3%, and 5%, respectively, for all patients. In Group A; the rates of stable disease; progressive disease; and partial response were at 55.4%, 41.9%, and 2.7%, respectively. The rates of LTP and IDR were 3.8%, and 63.5%, respectively, in Group B. Conclusion: TPCE is an effective treatment for colorectal lung metastases, which can be performed alone or combined with MWA.

## 1. Introduction

Colorectal carcinoma is counted among the most common types of cancer [1,2] and cancer-related deaths [1]. Furthermore, the most metastases from colorectal cancer occur and develop in the liver and lung [3].

The treatment of choice for colorectal lung metastases is still the surgical resection [4], but unfortunately, the majority of patients do not qualify for metastasectomy [5].

Systemic chemotherapy including 5-fluorouracil (5FU)/leucovorin with oxaliplatin (FOLFOX) or irinotecan (FOLFIRI) is usually applied for treating metastatic colorectal cancer [6]. High drug toxicity and side effects are considered upon the limitations of systemic chemotherapy [7].

In order to increase the concentration of anticancer drugs in the tumor itself, as well as to reduce and avoid systemic side effects, isolated lung perfusion can be performed [8,9].

The major disadvantages of ILP are the high invasiveness of this treatment option and that it can only be performed once per lung and not repetitively [10,11].

A less invasive treatment option for lung neoplasms is the transvenous pulmonary chemoembolization (TPCE), which can be performed repetitively and also allows the local application of chemotherapeutic agents and embolization materials [12].

TPCE is conducted by punction of the femoral vein and achieving a selective approach to the tumor-supplying arteries using different catheters. Subsequently, anticancer drugs can be applied and a stasis of the blood flow can be achieved by administration of lipiodol and microspheres [13]. The occlusion of the tumor-supplying arteries leads to an ischemic effect, but also prolongs the exposure time of the applicated cytostatics [12,14].

TPCE can be used as a palliative treatment to reduce the tumor burden [13,15], or as a neoadjuvant treatment option in combination with local ablation [15], which includes radiofrequency ablation (RFA) and microwave ablation (MWA), among others [16].

The purpose of this retrospective study was to evaluate local tumor response and survival of patients with unresectable and not to systemic chemotherapy-responding lung metastases from colorectal carcinoma by TPCE only as palliative treatment or by TPCE with an additional MWA therapy as potentially curative treatment.

## 2. Materials and Methods

### 2.1. Ethical Approval

This study received approval by the Ethics Committee of our university hospital.

### 2.2. Patients

This retrospective study included one hundred sixty-four (64 women and 100 men) patients with a mean age of 61.8 ± 12.7 years; median 64 years (range: 29–87 years). The patients had unresectable and not to systemic chemotherapy-responding pulmonary metastases from colorectal carcinoma that were either treated by only TPCE or TPCE followed by MWA, at our department of diagnostic and interventional radiology. The patients’ cases were discussed at a multidisciplinary tumor board (MTB), where the decision for the interventional treatments was made.

The unresectability of the metastases was stated by a thoracic surgeon in that MTB.

Most patients underwent only TPCE as palliative treatment to reduce tumor burden and symptoms, but also to at least achieve local tumor control. Patients who initially had a low number of metastases and responded well to TPCE also underwent MWA.

Most patients had their origin of primary tumor in the colon at a rate of 64% (105/164), followed by the rectum at a rate of 36% (59/164).

### 2.3. Palliative Group (Group A)

A total of 112 patients could be included in the palliative group and received only TPCE. The patient group consisted 48 women and 64 men with a mean age of 61 ± 13 years; median 62 years (range: 29–87 years).

Patients had predominant lung metastases with a mean metastatic count of 19 ± 19.7.

This group underwent an average of 5.6 ± 4 sessions of TPCE at intervals averaging four to six weeks during the course of therapy in a palliative therapeutic setting.

### 2.4. Potentially Curative Group (Group B)

This group consisted of 52 patients, including 16 women and 36 men, with a mean age of 63.5 ± 11.6 years and a median age of 66 years (range: 38–87 years), who were firstly treated with repetitive TPCE, and after reducing the size and number of metastases, received MWA with potential curative intent.

The patients initially had 6.6 ± 5.4 metastases and received a mean of 7 ± 5.5 sessions of TPCE at four- to six-week intervals. The patients were treated afterward by a mean of 3.4 ± 2.5 MWA sessions.

Table 1 summarizes the characteristics of patients and metastases.

### 2.5. Inclusion and Exclusion Criteria

We included patients who were (1) adults aged ≥18 years, (2) with unresectable and not to systemic chemotherapy-responding pulmonary metastases from colorectal cancer, and (3) with sufficient follow-up data, that underwent either repetitive TPCE as monotherapy or in combination with consecutive adjuvant MWA.

The exclusion criteria were (1) open arteriovenous shunt to the pulmonary venous system, (2) patients with thrombosis of the pulmonary artery, (3) cardiac, respiratory, and/or renal failure, (4) and coagulopathy.

### 2.6. TPCE Procedure

Informed consent was obtained from all patients before every treatment session.

Ahead of every treatment session, important laboratory parameters such as hemoglobin and creatinine levels, as well as platelet count, were checked.

After achieving local anesthesia with 1% mepivacaine, either the right or left femoral vein was punctured and a sheath was placed. A pulmonary angiographic overview was performed to assess the relevant tumor-supplying arteries. Afterward, the branches of pulmonary artery were selectively or super-selectively proceeded, followed by injection of a mixture of different chemotherapeutic agents. Different catheters were used while performing TPCE, including headhunter and cobra catheters.

A mixture consisting of mitomycin C 8 mg/m^2^ (mito-medac^®^, Medac), cisplatin 35 mg/m^2^ (Cisplatin Accord, Accord Healthcare Limited), and either gemcitabine 800 mg/m^2^ (Gemcitabin HEXAL^®^, Hexal AG) or irinotecan 100 mg/m^2^ (Irinotecan Aurobindo^®^, PUREN Pharma GmbH), in individual dosage was most frequently used.

**Figure 1:**
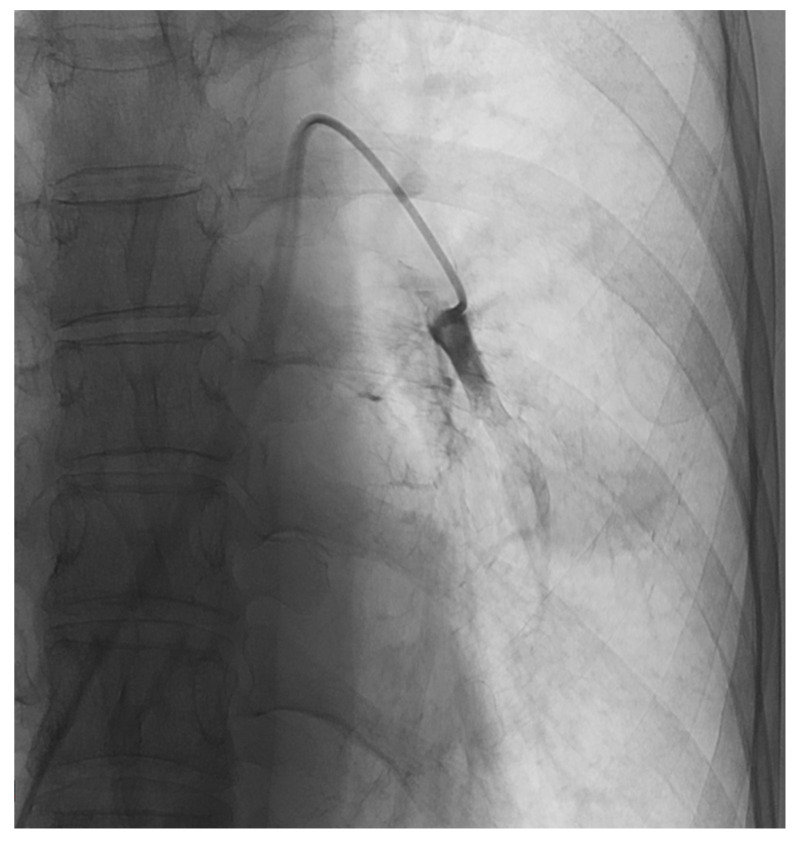
During TPCE of metastases in the left lung of a female patient.

Following the injection of chemotherapeutic agent, a stasis of the tumor blood supply was reached by applying up to 10 mL Lipiodol^®^ (Guerbert, Sulzbach, Germany) and 200–450 mg microspheres (EmboCept, PharmaCept GmbH, Berlin, Germany) gradually under fluoroscopy (Figure 1).

### 2.7. MWA Procedure

All relevant labor parameters were also verified before each MWA treatment.

**Figure 2:**
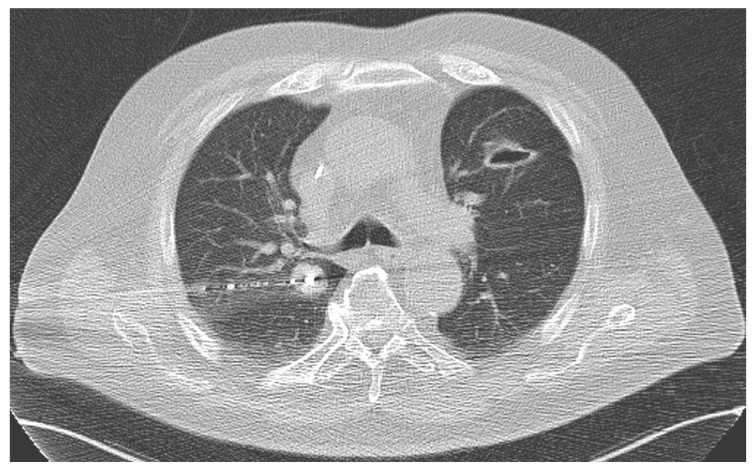
During MWA of a central metastasis (1.8 × 2.2 cm) in the right lung of a male patient.

At first, the target metastasis was again localized using an unenhanced CT scan. Next, the patient was covered in a sterile manner, disinfected, and the microwave antenna was placed in the intrapulmonary metastasis after local anesthesia. MWA treatments were conducted using the power of 45 to 100 watts within a duration of between 1 and 35 min (Figure 2).

### 2.8. Follow-Up

The patients received different cross-sectional scans before and after the different treatment sessions, including MRI and CT.

These pre- and post-interventional images were taken to evaluate the therapy response and to detect possible complications that may occur. These were also used to perform the measurements of the metastases and ablation areas. The volumetric measurements conducted were computer-aided.

### 2.9. Data Analysis

The cases were also evaluated according to different parameters, including age, sex, location of primary tumor, number of metastases, size (diameter and volume) of metastases, therapy response, and survival rates.

The revised response evaluation criteria in solid tumors (RECIST) was used to assess the treatment response [17] in Group A. A complete response (CR) was reached if the target lesions disappeared. A partial response (PR) was defined as a decrease in at least 30% of the target lesion. A progressive disease (PD) was displayed by an increase of at least 20%. The case was considered as a stable disease (SD), if it could not be allocated to PR or PD. The oncological response in Group B was divided into local tumor progression (LTP) and intrapulmonary distant recurrence (IDR).

The occurrence of new metastasis directly adjacent to the ablative margin was considered as LTP, and the development of new metastasis at any other site of the lung was evaluated as IDR [18].

Survival was calculated from the date of the treatment until the date of death or last contact. The survival analysis was performed for all patients, for Group A and Group B, as well as according to location of the primary tumor.

### 2.10. Statistical Analysis

We used IBM SPSS Statistics, Version 29.0 for performing the statistical analysis.

For calculation of survival rates the Kaplan–Meier estimator was used. The survival of subgroups was compared between the groups with a log-rank test.

We considered a two-sided *p*-value of ≤5% as statistically significant.

## 3. Results

### 3.1. Measurements of Metastases and Ablation Area

The mean diameter of the reference metastases was 2.3 ± 1.7 cm and the mean volume was initially 20.4 ± 49.2 cm^3^ in Group A. In Group B, the mean diameter of the reference metastases was 1.9 ± 1.5 cm and the mean volume was 9.3 ± 20.7 cm^3^, initially.

The mean volume of metastases was reduced after TPCE to 7.9 ± 15.5 cm^3^ before performing the MWA treatment.

The mean volume of the ablation area was 45.4 ± 35 cm^3^.

### 3.2. Therapy Response

In Group A, 55.4% (*n* = 62) of the patients achieved SD, and 47 patients had PD at a rate of 41.9%. PR was achieved in 2.7% (*n* = 3) of the patients (Table 2).

A total of 35 patients developed a recurrence at a rate of 67.3% in Group B.

Of these patients, two cases developed LTP at a rate of 3.8%, and 33 patients developed IDR at a rate of 63.5% (Table 3).

### 3.3. Overall Survival

**Figure 3:**
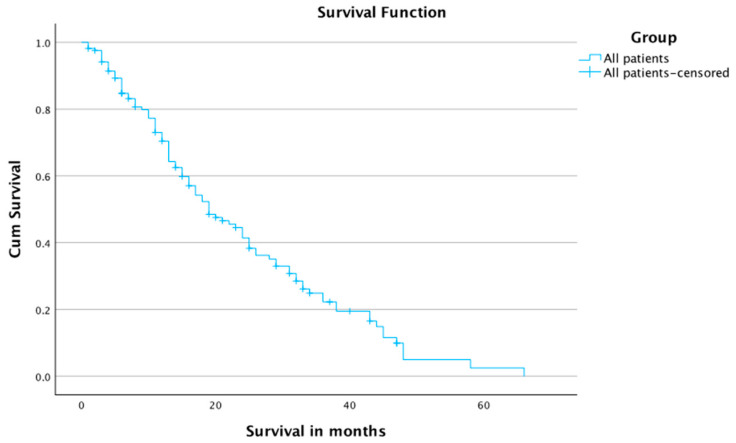
Survival for all patients.

The 1-, 2-, 3-, and 4-year survival rates were 70.4%, 41.4%, 22.3%, and 5%, respectively, for all patients. The mean and median times of survival for all patients were 23.7 months and 19 months, respectively (Table 4 and Figure 3).

The subgroup analysis of survival showed 1-, 2-, and 3-year survival rates of 61.2%, 33.5%, and 17%, in Group A, and 87%, 55.3%, and 31.1% in Group B, respectively.

**Figure 4:**
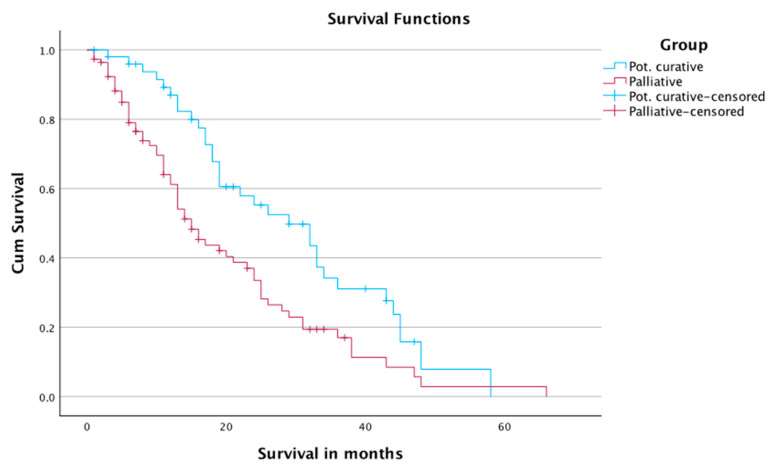
Survival of patients according to treatment intent.

The mean survival time was 20 months in Group A, and 30 months in Group B. The median survival times in Group A and Group B were 15 months and 29 months, respectively (Table 5 and Figure 4). The number of censored cases was 46 in Group A and 19 in Group B.

**Figure 5:**
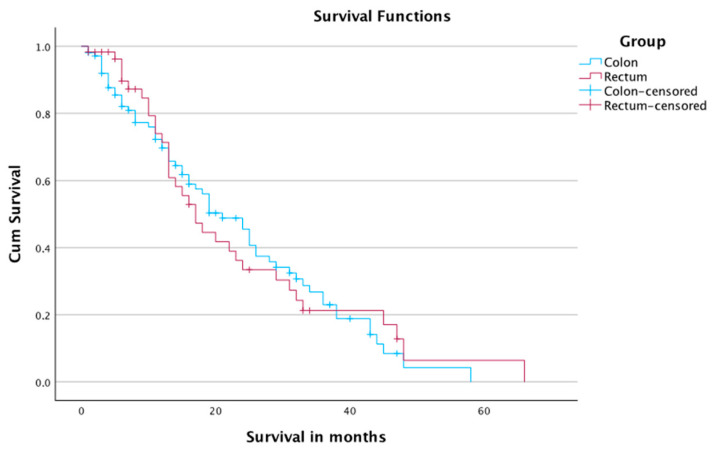
Survival of patients according to origin of primary tumor.

There was no significant difference in the survival of the patients when comparing it according to location of primary (*p*-value: 0.85). The 1-, 2-, and 4-year survival rates were 69.7%, 45.5%, and 4.2% in patients with colon carcinoma, and 71.4%, 33.4%, and 6.4% in patients with rectal carcinoma, respectively. The median survival time was 21 months in patients with colon carcinoma, and 17 months in patients with rectal carcinoma (Table 6 and Figure 5). A total of 40 cases with colon carcinoma and 25 cases with rectal carcinoma were censored.

## 4. Discussion

Unfortunately, metastases occur in more than half of the patients with colorectal carcinoma throughout the time of their disease [19]. This shows the importance of suitable therapeutic management for the metastases in patients with colorectal carcinoma.

Unfortunately, there is still not enough data and publications in the literature that evaluated the minimally invasive local therapies such as TPCE and MWA for colorectal lung metastases in different treatment intents.

For this reason, the current study focused on the efficacy of two locoregional treatments of colorectal lung metastases with two different intentions. Group A received TPCE alone as palliative therapy, and Group B was treated by TPCE to reduce the size and number of metastases, and after that, MWA was performed in potentially curative setting.

The patients in Group A had initially a high mean metastatic count of 19. The patients in Group B had an initially lower mean metastatic count of 6.6.

We found that these treatments for colorectal lung metastases provide good local tumor control and satisfactory overall survival.

Survival of the patients with lung metastases could be prolonged by systemic chemotherapy, but this therapy option is usually limited by some factors, including resistance to the drugs administered among others [20].

Using isolated lung perfusion, a higher concentration of chemotherapeutic agents can be reached in the tumor compared to intravenously administered systemic chemotherapy [14,21]. However, the procedure is very invasive, as it is usually performed via thoracotomy [22,23], which represents a high physical burden for the patients.

TPCE shows some advantages over systemic chemotherapy and isolated lung perfusion.

By performing TPCE, a high localized concentration of anticancer drugs can be reached in the tumor without relevant systemic side effects [14], which makes TPCE more well-tolerated than systemic chemotherapy. The main benefit of TPCE over isolated lung perfusion is that it is less invasive and can be repeatedly performed by selective application of the chemotherapeutic agents and embolization materials [14].

Even surgical resection of lung metastases is still the gold standard, which shows very promising results (5-year survival rate up to 56%) [24], only about 30% of patients are suitable for metastasectomy [25].

The are several published studies on thermal ablation of colorectal lung metastases, which mainly focus on evaluation of the outcome of patients who underwent RFA [26,27,28] or MWA [29,30,31].

Yamakado et al. [26] evaluated the RFA of colorectal lung metastases in 78 patients with 198 lesions on a long-term basis. They reported 1- and 3-year survival rates at 83.9% and 56.1%, respectively. The median survival time was 38 months. Our 1- and 3-year survival rates were 87% and 31.1%, respectively, and the median survival time was 29 months in Group B (TPCE and MWA).

There are few previous publications that investigated TPCE of primary [13] and secondary lung tumors [15,32]. Vogl et al. [13] included 17 patients who had primary tumors including adenocarcinoma, pleural mesothelioma, squamous cell carcinoma, small cell carcinoma, and non-small cell carcinoma, and were treated by TPCE. Therapy response was observed in 23.5% of the cases. The rates of SD and PD were 41.2% and 35.3%, respectively. Our response in Group A (TPCE only) was noticeably lower at 2.7%. Our rates of SD at 55.4% and PD at 41.9% were higher. The mean and median survival times were 405.9 days and 394 days, respectively. Our current mean and median survival times were longer.

Another study included 52 patients with 106 unresectable lung metastases, mainly from colorectal carcinoma, breast cancer, renal cellular carcinoma, thyroid cancer, cholangiocellular carcinoma, and leiomyosarcoma, that underwent TPCE [32]. They reported a mean survival time of 17 months and a median survival time 21.1 months. These survival times are comparable with our results.

A total of 16 patients at a rate of 30.7% showed a response, which is higher than our rate in the TPCE group of this study. A total of 13.5% (*n* = 7) of the patients achieved SD, and 29 patients developed PD at a rate of 55.8%. Our rate of SD was higher and the rate of PD was lower in the recent study.

Pulmonary metastases from HCC can be also treated by locoregional treatments [33,34].

Duan et al. [33] enrolled in their study a total of 52 HCC patients with lung metastases, who were treated by sorafenib, transcatheter arterial chemoembolization, and bronchial transarterial chemoinfusion. They reported a median overall survival time of 12 months.

Hori et al. [34] included 14 patients with mediastinal or pulmonary metastases from hepatocellular carcinoma (HCC), who were treated with transarterial chemoembolization. They reported a median survival time of 15 months, and 1-, 2-, and 3-year survival rates at 57.1%, 28.6%, and 19.1%, respectively. The subgroup analysis of survival showed 1-, 2-, and 3-year survival rates of 61.2%, 33.5%, and 17%, in Group A. These results are very similar with our results in Group A.

This study had several limitations. The first limitation of this study was its retrospective design. Secondly, this study was performed at a single institution. Thirdly, the impact of some factors, including the mutation status of colorectal cancer, which potentially has an impact on the survival of the patients, were not considered. Fourthly, the real effect of TPCE on OS remains unclear, as we did not include a control group without TPCE. Lastly, important data and parameters, such as tumor marker, were not evaluated. Additionally, the safety and possibly occurring complication were not investigated. A prospective study that includes all important data as well as a control group to accurately investigate the efficacy and safety of TPCE and MWA of colorectal lung metastases should be conducted.

## 5. Conclusions

We showed in this study, that TPCE and MWA are effective and suitable treatments for unresectable and not to systemic chemotherapy-responding lung metastases from colorectal carcinoma. The patients that only underwent TPCE in palliative intent had satisfactory survival and achieved good local tumor control.

TPCE can be also applied as a neoadjuvant procedure in combination with thermal ablation such as MWA. The combination of TPCE and MWA was also effective in curative therapeutic intent and provided a promising survival time.

The subgroup analysis did not show a significant difference in the overall survival between patients with colon carcinoma and rectal carcinoma.

## Figures and Tables

**Table 1:** Patients and metastases.

	Group A + B	Group A	Group B
Number of patients (%)	164 (100)	112 (68.3)	52 (31.7)
Gender *n* (%)			
Women	64 (39)	48 (42.9)	16 (30.8)
Men	100 (61)	64 (57.1)	36 (69.2)
Mean age	61.8 ± 12.7 years	61 ± 13 years	63.5 ± 11.6 years
Primary origin *n* (%)			
Colon	105 (54)	72 (64)	33 (63.5)
Rectum	59 (36)	40 (36)	19 (36.5)
Mean number of metastases		19 ± 19.7	6.6 ± 5.4
Initial diameter of metastases		2.3 ± 1.7 cm	1.9 ± 1.5 cm
Initial volume of metastases		20.4 ± 49.2 cm^3^	9.3 ± 20.7 cm^3^
Number of TPCE sessions		5.6 ± 4	7 ± 5.5
Number of MWA sessions			3.4 ± 2.5

**Table 2:** Therapy response of Group A.

Stable disease *n* (%)	62 (55.4)
Progressive disease *n* (%)	47 (41.9)
Partial response *n* (%)	3 (2.7)

**Table 3:** Therapy response of Group B.

LTP *n* (%)	2 (3.8)
IDR *n* (%)	33 (63.5)

**Table 4:** Survival of all patients.

1-year OS rate.	70.4%
2-year OS rate	41.4%
3-year OS rate	22.3%
4-year OS rate	5%
Mean OS time	23.7 months
Median OS time	19 months

**Table 5:** Survival of patients according to treatment intention.

	Group A	Group B
1-year OS rate	61.2%	87%
2-year OS rate	33.5%	55.3%
3-year OS rate	17%	31.1%
Mean OS time	20 months	30 months
Median OS time	15 months	29 months

**Table 6:** Survival of patients regarding to cancer origin.

	Colon	Rectum
1-year OS rate	69.7%	71.4%
2-year OS rate	45.5%	33.4%
4-year OS rate	4.2%	6.4%
Median OS time	21 months	17 months
*p*-Value	0.85	

## Data Availability

Data is available on request from the corresponding author.
